# Genomic Influence in the Prevention of Cardiovascular Diseases with a Sterol-Based Treatment

**DOI:** 10.3390/diseases6020024

**Published:** 2018-04-03

**Authors:** Ismael San Mauro Martín, Javier Andrés Blumenfeld Olivares, Eva Pérez Arruche, Esperanza Arce Delgado, María José Ciudad Cabañas, Elena Garicano Vilar, Luis Collado Yurrita

**Affiliations:** 1 Research Centers in Nutrition and Health, Paseo de la Habana, 28036 Madrid, Spain; elena@grupocinusa.es; 2 Hospital El Escorial, San Lorenzo de El Escorial, 28200 Madrid, Spain; javierandres.blumenfeld@salud.madrid.org (J.A.B.O.); eva.perez@grupocinusa.es (E.P.A.); e.arcedelgado@gmail.com (E.A.D.); 3 Department of Medicine, Universidad Complutense de Madrid, 28040 Madrid, Spain; mjciudad@ucm.es (M.J.C.C.); lcollado@med.ucm.es (L.C.Y.)

**Keywords:** genetic, nutrigenetics, sterol, cholesterol, low-density lipoprotein cholesterol, cardiovascular disease

## Abstract

Raised serum cholesterol concentration is a well-established risk factor in cardiovascular disease. In addition, genetic load may have an indirect influence on cardiovascular risk. Plant-based sterol-supplemented foods are recommended to help reduce the serum low-density lipoprotein cholesterol level. The objective was to analyse the influence of different polymorphisms in hypercholesterolemia patients following a dietary treatment with plant sterols. A randomised double-blind cross-over controlled clinical trial was carried out in 45 people (25 women). Commercial milk, containing 2.24 g of sterols, was ingested daily during a 3-week period, and then the same amount of skim milk, without sterols, was consumed daily during the 3-week placebo phase. Both phases were separated by a washout period of 2 weeks. At the beginning and end of each phase, blood draws were performed. Genes *LIPC C-514T* and *APOA5 C56G* are Ser19Trp carriers and greatly benefit from sterol intake in the diet. *LIPC C-514T* TT homozygous carriers had lower low-density lipoprotein cholesterol (LDL-c) levels than CC homozygote and CT heterozygote carriers after the ingestion of plant sterols (*p* = 0.001). These two genes also showed statistically significant changes in total cholesterol levels (*p* = 0.025; *p* = 0.005), and no significant changes in high-density lipoprotein (HDL) cholesterol levels (*p* = 0.032; *p* = 0.003), respectively. No statistically significant differences were observed for other genes. Further studies are needed to establish which genotype combinations would be the most protective against hypercholesterolemia.

## 1. Introduction

Human health is a result of complex interactions between genetic predisposition and the environment in which genes manifest. It has long been recognised that individual differences in genetic variation influence the association between dietary recommendations and health, which is yet to be reflected in the dietary guidelines. Identifying the interplay between genes and dietary patterns holds promise for a new era of personalised medicine, whereby the recommended diet for best health is tailored towards how an individual’s metabolism is genetically predisposed to respond to dietary intake [1].

The understanding of genetic approaches in the nutritional sciences is referred to as nutrigenomics. Nutrigenomics explores the interaction between genetic factors and dietary nutrient intake regarding various disease phenotypes and general health [2], with the aim to provide more personalised dietary advice [3,4].

Through the use of genome-wide association studies, genetic variations (single nucleotide polymorphisms) have been identified as genetic factors, making it more likely to determine individual disease predisposition [2].

Naturally occurring variants of the apolipoprotein A5 gene have been associated with increased triglyceride levels, and have been found to confer risk for cardiovascular diseases. Some of the *MTHFR* gene polymorphisms are also associated with an increased risk of congenital heart failure [5] and cardiovascular disease [6], especially the C677T genetic polymorphism [7]. The hepatic lipase gene (LIPC) is responsible for the hydrolysis of triglycerides [8]. Genetic studies and numerous epidemiologic studies have identified Lp(a) as a risk factor for atherosclerotic diseases, such as coronary heart disease and stroke [9], as it is related to low-density lipoprotein cholesterol (LDL-c) [10]. In addition, the genetic load may have an indirect influence on cardiovascular risk. ApoE plays an essential role in the catabolism of lipoproteins [11].

Cholesterol metabolism is a well-defined responder to dietary intake, and a classic biomarker of cardiovascular health. For this reason, circulating cholesterol levels have become essential in shaping the nutritional recommendations of health authorities worldwide for better management of cardiovascular disease, a leading cause of mortality and one of the most costly health problems globally [12].

A number of cholesterol-related gene–diet interactions have been confirmed, some of which may have clinical importance, supporting a deeper insight into the rapidly emerging field of nutrigenetics for meaningful conclusions that may eventually lead to genetically-targeted dietary recommendations, in the era of personalised nutrition [12].

On the other hand, nutrition and dietary treatments are important in managing cardiovascular disease. Plant sterols have been postulated as beneficial regulatory agents for the control of the disease [13,14,15]. The daily consumption of phytosterol-enriched foods is widely prescribed as a therapeutic option to reduce LDL-c levels in plasma, and therefore reduce the risk of atherosclerotic disease [16].

Experts from the European Food Safety Authority confirmed that blood cholesterol can be reduced by an average of 7–10.5% if a person consumes 1.5–2.4 g of plant sterols a day. This effect is generally observed within the first 2–3 weeks. Mid- and long-term studies, of up to 85 weeks, show that the blood cholesterol reducing effect could be sustained throughout that period [17].

Future studies should be carried out, with the aim of learning about the increasing difference and soundness of dietary treatment data available in concordance with individual genomic profiles.

The aim of this study was to analyse the influence of different polymorphisms in hypercholesterolemia patients following dietary treatment with plant sterols.

## 2. Materials and Methods

A randomised double-blind cross-over controlled clinical trial was designed. Participants were recruited at the San Carlos Clinical University Hospital in Madrid (SCCUHM) and the El Escorial Hospital in Madrid. Volunteers throughout the hospital were invited to participate. Patients from primary care and endocrinology departments were specifically invited to participate. Prior to the trial, the participants received instructions on the purpose of the study and signed an informed consent form.

The plant sterols were ingested using Naturcol milk (supplied by *Corporación Alimentaria Peñasanta, S.A.*, Asturias, Spain), available on the market throughout the whole study. Treatment and placebo skim milk were identical in sensorial and nutrient composition.

**Figure 1:**
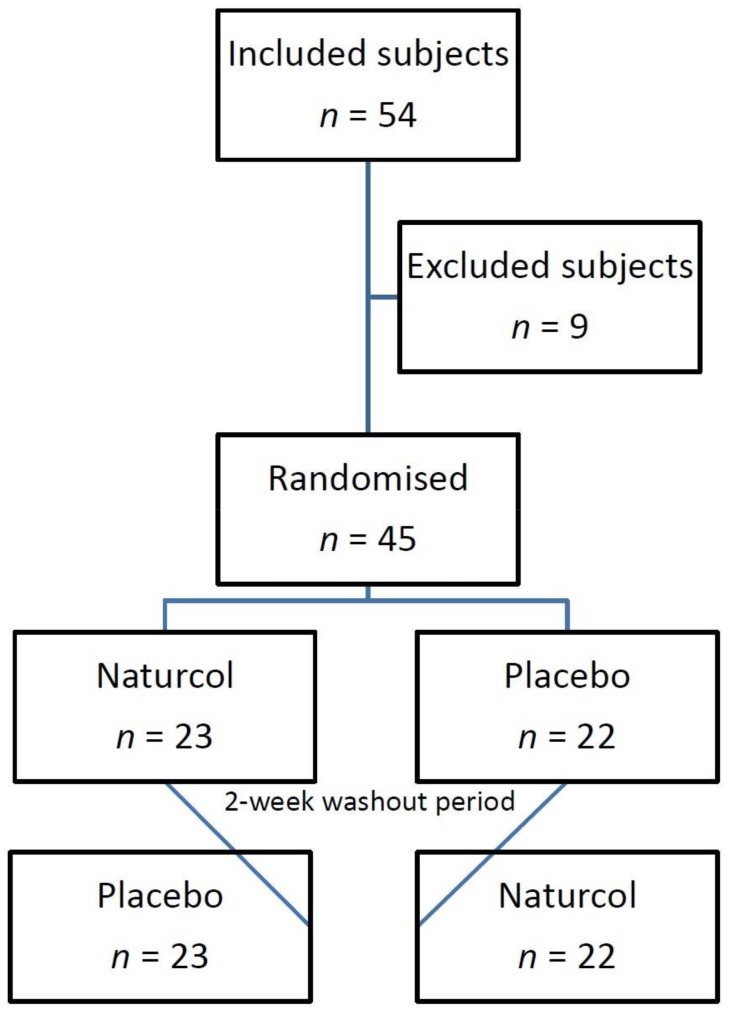
Flowchart of the study participants.

In this crossover study, all subjects participated in both groups, with two phases of three weeks of each treatment, separated by a two-week washout period (Figure 1). A 2-week washout system was implemented to take into account the metabolism and excretion process, and the 2-day functionality of the sterol in the human body [18].

Blood samples were taken at the beginning and at the end of each phase. Over a three-week period, the subjects ingested two glasses of either type of milk daily. Each glass had a standard capacity of 350 mL. Plant sterols were administered daily through the skim milk in quantities of 2.24 g (in plant sterol phase), the same quantity of skim milk without plant sterol was used for the placebo. An 80% target was set as the minimum threshold for consumption. Milk was packaged without labelling to ensure neither the subjects nor the researchers were aware of the type of milk, and were only differentiated by the lid’s colour. Groups were randomly assigned, using random number tables.

### 2.1. Sample Size

Of the 54 initial participants, 45 completed the genetic study: 25 women and 20 men with a mean age of 37.9 ± 7.5 years. The sample was recruited based on total cholesterol as the primary biomarker, according to the standard formulas for that purpose. The formula was calculated for a difference of ±15% of total cholesterol (200 mg/dL), taking into account a standard deviation of 35 mg/dL for a confidence interval of 95%, a statistical power of 85%, and a loss rate of 10%, using the formula for sample size. 

### 2.2. Inclusion and Exclusion Criteria

Inclusion criteria: men and women, aged 18–50 years, with total cholesterol (TC) > 200 mg/dL.

Exclusion criteria: TC < 200 mg/dL, cardiac pathology (myocardial infarction, angina); lactose intolerance, allergy to cow’s milk proteins or plant sterols; obesity (body mass index –BMI– >30) or pharmacological treatment (for cholesterol or fibrate triglycerides, statins, etc.). Subjects that already supplemented with plant sterols. None of the participants presented active thyroid disease, liver disease, alcoholism, or any other such condition that dynamically altered lipid levels and/or diets. 

### 2.3. Clinical Analyses

The samples for the analytical tests were extracted by medical staff after a 12-h fast at the Clinical Analysis Unit in the SCCUHM and El Escorial Hospital, and in the San Carlos Specialty Unit in San Lorenzo de El Escorial.

The blood extraction protocol of the laboratory was as follows: blood extraction was performed with a gel serum tube, using the technique ‘Blood collection’ with an s-Monovette in aspiration [19,20,21]. After extraction, the samples were kept at 5 ± 3 °C until their arrival at the laboratory. The centrifugation settings were T 20 ± 5 °C, for 10 minutes, at 1200 g. Stability: 1 week at 5 ± 3 °C. The Ultraviolet–Visible spectrophotometry technique was used.

The laboratory that performed the tests is located in the same city of the study, and has the required legal accreditations, certificates, and standards, ISO 9001:2008, and accreditation 511/LE2114 according to criteria included in the UNE-EN ISO 15189 Standard.

### 2.4. Genetic

A variation in the effect of dietary treatment with plant sterols in milk on lipoprotein metabolism (reduction of LDL-c) was observed; therefore, a genomic analysis was performed.

With this analysis, we sought to understand the following hypothesis: (1) that genetic polymorphisms could be associated with a higher or lower response effect to the plant sterol treatment for hypercholesterolemia. 

Polymorphisms APOA5 C56G Ser19Trp, Prothrombin G20210A, F5 Arg506Gln, MTHFR C677T, LIPC C-514T, LPA I4300M, PPAR_ALPHA L162V, APOA5 1131T>C, APOE APOE2/3/4 and APOE APOE2,3,4 were studied.

Genomic DNA, for genotyping the SNP, was extracted from saliva samples. The analyses were run by Vitagenes Lab (San Francisco, CA, USA). The genotyping was conducted using the Biobank Axiom1 96-Array from Affymetrix. Genotype calling was performed with respect to Affymetrix’s best practice guidelines, including analysis with SNPolisher, assuming a quality control rate of >0.97 [22,23].

The extraction and purification of DNA from saliva samples was carried out as follows: each DNA extraction was run on an agarose gel to ensure high quality and high molecular weight. To ensure maximum purity of the extracted DNA (ratio > 1.7), the OD260/280 ratio was analysed, that is, the optical density of the extracted DNA at 260 nm and 280 nm wavelengths. All analyses were performed in duplicate to ensure maximum precision and reliability in their results.

All Vitagenes analytical processes were carried out in laboratories that had CLIA certification, thus attaining the optimal level of technical quality and precision proposed by the FDA in the United States [24].

### 2.5. Variables and Study Factors

An ad hoc questionnaire was designed for the study. The questionnaire and anthropometric study were performed by a single trained researcher, ensuring the homogeneity and standardisation of the uniformity criteria and methodology to follow. The study variables were established in terms of the proposed objectives: sex, age, clinical and pharmacological history, sleep quality, health habits, use of tobacco and alcohol, intestinal transit, food consumption frequency, and physical activity. In addition, each participant’s weight, height, waist perimeter, BMI, fat percentage, subcutaneous fat percentage, and lean body mass (kg) were measured. Weight, BMI and body composition were determined by means of tetrapolar multi-frequency (20 and 100 kHz) electrical bioimpedance, InBody Model 230, following the usual standard protocol and the manufacturer’s recommendations [25]. Waist perimeter was measured with a flexible non-elastic metal measuring tape with a range of 0.1 mm–150 cm. Basal metabolic rate was calculated with the following formulas: BMR = 66 + (13.75 × weight in kg) + (5 × height in cm) − (6.8 × age in yrs), for men; BMR = 655 + (9.6 × weight in kg) + (1.8 × height in cm) − (4.7 × age in Yrs), for women. The analytical markers were as follows: lipid profile (total cholesterol, high-density lipoprotein cholesterol (HDL-c), LDL-c, and non-HDL-cholesterol. Confounding factors were also taken into account with an affinity table after ingestion (>95%), monitoring of the non-modification of baseline habits during the trial, and a record of food consumption frequencies; in order to control the ingestion of foods that may influence the metabolism of cholesterol upwards or downwards.

### 2.6. Statistical Analysis

The data were analysed using the SPSS 21.0 statistical package. A descriptive analysis was first made of the sociodemographic and anthropometric data, the baseline, and the final lipid values under the ingestion of Naturcol and the placebo. The normality of the lipid values was determined using the Shapiro–Wilk test. To analyse the efficacy of the ingestion of Naturcol and the placebo, the difference in lipid values was calculated before and after ingestion, also applying the Student’s *T*-test, for related samples, or the Wilcoxon rank sum test according to the compliance with the assumption of normality of the dependent variables. The efficacy of the intervention was verified by comparing the differences (final-baseline) in the ingestion of milk with sterols and the placebo, by applying the Student’s *T*-test for related samples, or the Wilcoxon rank sum test depending on the compliance with the assumption of normality of the lipid increase. The effect size and the proportion of the mean differences were calculated with regard to the standard deviation of the baseline, or milk with sterols as the case may be. The level of significance applied was 5%.

The study was approved by the respective bioethics committees of the San Carlos Clinical University Hospital in Madrid (SCCUHM) and the El Escorial Hospital in Madrid. 

This study followed the ethical principles enshrined in the Helsinki Declaration, the recommendations for good clinical practice, current Spanish legislation regulating clinical research in humans, and the protection of personal and bioethical data (Royal Decree 561/1993 on clinical trials and 14/2007, 3 July for biomedical research).

## 3. Results

A total of 45 subjects (25 women and 20 men) completed the trial. Nine subjects failed to complete the trial because of different genotyping, and failure to complete the plant sterols treatment (ingested less than 80% of the consumption protocol). They had an average age of 37.9 ± 7.5 years and weighed 69 kg (BMI 23.7 kg/m^2^) (Table 1). Baseline total cholesterol was 236.6 mg/dL, LDL cholesterol was 157.3 mg/dL and HDL cholesterol was 58.2 mg/dL. There were no differences between centres in volunteer demographics. In Table 2, the descriptive statistic of genes and haplotypes can be found.

**Figure 2:**
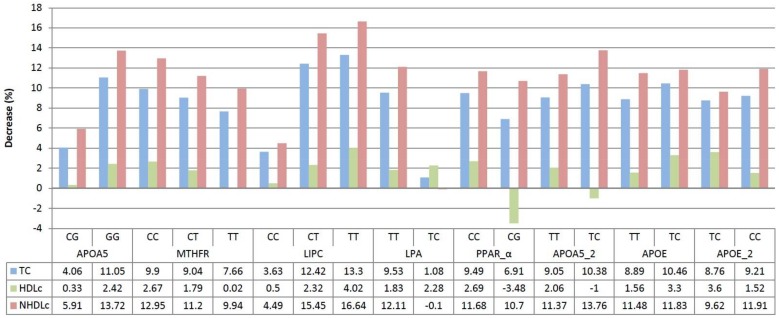
Percentage difference in lipid parameters before treatment, based on genes and haplotypes. TC: total cholesterol; HDLc: high density lipoprotein cholesterol; NHDLc: non-high density lipoprotein cholesterol.

No statistically significant differences (*p* > 0.05) were found between the decreased percentage of lipid parameters (total cholesterol, high-density lipoprotein cholesterol (HDL) cholesterol, and Non-HDL cholesterol), and the genotypes observed for the following genes (Figure 2): MTHFR C677T, LPA I4300M, PPAR_ALPHA L162V, APOA5 1131T>C, APOE APOE2/3/4 (*p* = 0.416) and APOE APOE2,3,4.

Only APOA5 C56G Ser19Trp and LIPC C-514T genes incurred statistically significant changes for levels of total cholesterol (*p* = 0.025; *p* = 0.005), but not HDL cholesterol (*p* = 0.032; *p* = 0.003), respectively, after the treatment with sterols (Figure 2).

**Figure 3:**
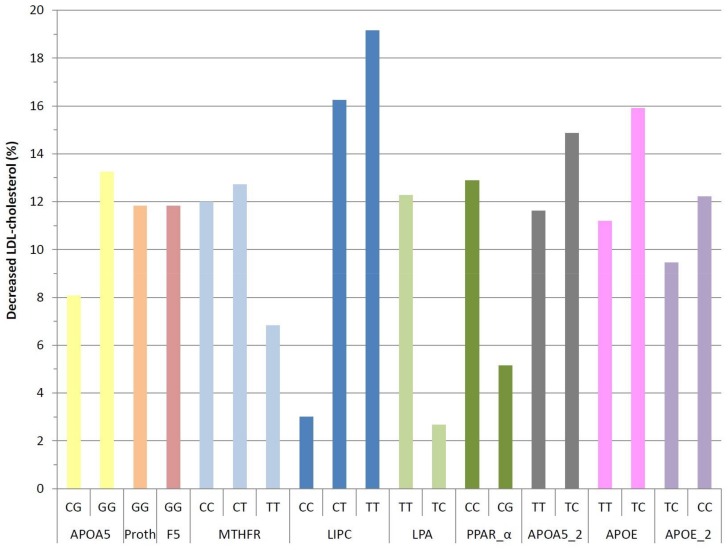
Percentage difference in low-density lipoprotein (LDL) cholesterol values before treatment, according to genes and haplotypes.

LIPC C-514T, APOA5 1131T>C and APOE APOE2/3/4 greatly benefit from sterol intake in the diet (Figure 3). In LIPC C-514T carriers, TT homozygous and CT heterozygote carriers lowered their LDL-c more than CC-homozygote carriers, after the ingestion of plant sterols (*p* = 0.001). APOA5 1131T>C and APOE APOE2/3/4 did not show statistically significant differences (*p* = 0.682; *p* = 0.416).

A large difference was also observed in the case of LPA I4300M and PPAR_alpha L162V genes, between the carriers of the homozygous (TT and CC, respectively) and heterozygous (TC and CG, respectively) variants, although the difference is not statistically significant (*p* = 0.121; *p* = 0.180).

Polymorphisms APOA5 C56G Ser19Trp, MTHFR C677T and APOE APOE2,3,4 exert a favourable effect in the treatment, but no statistically significant differences in the reduction of LDL-c can be seen (*p* = 0.246; *p* = 0.662; *p* = 0.637).

The analytical study could not be applied to Prothrombin G20210A and F5 Arg506Gln genes, because each gene had only one haplotype.

After checking whether any genotype was directly related to baseline LDL-c, it was found that only one of the analysed genes (APOA5 C56G Ser19Trp; *p* = 0.013) was associated with different rates of baseline cholesterol, according to their single nucleotide polymorphism (SNP).

Other genes and their haplotypes could not be linked to a higher or lower level of basal cholesterol, as measured by the parametric test, *T*-Student test or ANOVA. This means a possible protection of this risk factor (LDL-c) could not establish by genotype. The obtained results for each gene were as follows: MTHFR C677T (*p* = 0.184), LIPC C-514T (*p* = 0.522), LPA I4300M (*p* = 0.158), PPAR_ALPHA L162V (*p* = 0.936), APOA5 1131T>C (*p* = 0.484), APOE APOE2/3/4 (*p* = 0.604), and APOE APOE2,3,4 (*p* = 0.233).

## 4. Discussion

Phytosterols are known to reduce serum LDL-c level without changing HDL-c levels. Daily consumption of phytosterol-enriched foods is widely used as a therapeutic option to lower plasma cholesterol and the risk of atherosclerotic disease [26]. Clifton et al. (2004) [27], who analysed the differences of using plant sterols in different matrices, concluded that the matrix that had a better effect in reducing LDL-c was obtained in milk.

When the recent scientific literature is reviewed, important studies [28] evaluating the benefit of plant sterol consumption on cardiovascular risk biomarkers can be found, as a meta-analysis of more than 40 clinical trials concluded [29].

Like other authors [29,30], this study analysed whether the effectiveness of plant sterols is dependent on the subjects’ baseline levels; finding a higher decrease in LDL-c, the greater baseline, depends on the subjects’ innate genes. Lipid metabolism, of great importance regarding cardiovascular risk, is regulated by many genes whose variants may influence this risk [11].

Maasz et al. (2008) [31] suggested that the 56G allele can confer risk exclusively for the development of large-vessel associated stroke. Thereby, the 56G allele differs from the APOA5T-1131C allelic variant, which has been previously identified as a risk factor for all subgroups of the stroke disease, namely the C allele variant, which is a risk factor for heart disease and ischemic stroke [32,33], by increasing levels of triglycerides. This polymorphism has a significant association with coronary heart disease risk [34]. Sanchez-Moreno et al. (2011) [35] found that very low-density lipoprotein cholesterol (VLDL-C) concentrations were higher in carriers of the minor allele than in noncarriers (0.7 ± 0.32 vs. 0.6 ± 0.22 mmol/L) (*p* = 0.01).

Regarding the ApoE, it has been observed that the ε4 allele of the ApoE4 is a major risk factor for coronary heart disease. However, the same results were not observed in the case of ApoE2, whose polymorphisms did not seem significantly related to coronary risk [36].

The relationship between the hepatic lipase gene and the influence on HDL-c has not been clearly established, although it seems to be an inverse relationship [8]. The influence that this gene has on the metabolism of glycerophospholipids [37] may also alter plasma concentrations thereof. The results published by Posadas-Sanchez et al. (2015) [38] suggest that the LIPC C-154T polymorphism is associated with cardiometabolic parameters and cardiovascular risk factors: under the dominant model, the TT genotype was associated with increased levels of the triglycerides/HDL-cholesterol index (*p* = 0.046). On the other hand, the same genotype was associated with the presence of small LDLs (*p* = 0.003). The risk analysis showed that under a dominant model, the LIPCC-514T polymorphism was associated with increased hypertriglyceridemia (OR = 1.36, *p* = 0.006) and coronary artery calcification (OR = 1.44, *p* = 0.015). The T allele carriers had higher levels of low-density lipoprotein cholesterol (LDL-C) in obese boys, and TC and LDL-C in non-obese girls (all *p* < 0.05) [39].

Li et al. (2015) [36] exposed that for the C677T polymorphism, when compared with the wild CC genotype, heterozygosity of CT increased the risk of congenital heart disease (OR = 2.249, 95% CI 1.305–3.877, *p* = 0.003), and the homozygous mutant genotype TT was significantly associated with the risk of congenital heart disease (OR = 3.121, 95% CI 1.612–6.043, *p* = 0.001).

The results suggested that genetic and metabolic biomarkers when used together may predict inter-individual lipid level responsiveness to the plant-sterol-intervention, and thus could be useful in devising individualised cholesterol-lowering strategies. We cannot find an accurate and concise explanation, beyond the observation of these differences in the results, which should undoubtedly be refuted until more research can corroborate our findings, and with samples of the wider population. 

However, nutrigenomics has already observed these differences for years, as mutations in some genes are known to hinder gene function, for example, in the classic studies of PUFAS and Framingham [40,41,42]. It has not supposed a clinical transcendence as suggestive, as revealed by its important results.

## 5. Conclusions

Having analysed the influence of different polymorphisms in hypercholesterolemia patients following a dietary treatment with plant sterols, we can conclude that gene LIPC C-514T carriers, especially TT homozygous carriers, lowered LDL-c more than CC homozygote and CT heterozygote carriers, after ingestion of plant sterols. LIPC C-514T and APOA5 C56G Ser19Trp carriers greatly improve their lipid profile from sterol intake in the diet. No statistically significant differences were observed for other genes.

Human genome discoveries need to be moved into health practice in a way that maximises health benefits and minimises harm to individuals and populations.

These results provide the basis for further studies to establish which genotype combinations would be the most protective against hypercholesterolemia.

## Figures and Tables

**Table 1:** Descriptive statistics of the anthropometric measurements and lipid profile.

	Total (*n* = 45)	Males (*n* = 20)	Females (*n* = 25)
	Mean ± SD	Mean ± SD	Mean ± SD
Age (years)	37.9 ± 7.5	39.7 ± 6.9	36.5 ± 7.8
Weight (kg)	68.6 ± 12.4	77 ± 11.3	61.4 ± 8.03
Height (m)	1.7 ± 0.1	1.76 ± 0.1	1.64 ± 0.1
BMI (Kg/m^2^)	23.7 ± 3.1	24.8 ± 3.2	22.8 ± 2.7
Body fat (%)	25.5 ± 7.3	21.2 ± 6.5	29.4 ± 5.7
Visceral fat (kg)	6.8 ± 4.6	8 ± 4.7	5.7 ± 4.3
Muscle (kg)	35.5 ± 13.4	40.4 ± 14.1	31.2 ± 11.4
Basal metabolic rate (kcal)	1484.5 ± 278.6	1705 ± 230.1	1285 ± 125.3
	Decrease % (from baseline to final measures)		
Total cholesterol (mg/dL)	9.1 ± 9.3	8.2 ± 9.8	9.9 ± 9.3
LDL-cholesterol (mg/dL)	11.8 ± 13.04	9.9 ± 12.9	13.4 ± 13.8
HDL-cholesterol (mg/dL)	1.8 ± 8.1	2.1 ± 6.9	1.5 ± 9.3
Non-HDL-cholesterol (mg/dL)	11.5 ± 10.8	9.6 ± 11.7	13.2 ± 10.3

**Table 2:** Descriptive statistics of genes and haplotypes.

Gene	Haplotype	Frequency (*n*)	Percentage (%)
APOA5 C56G Ser19Trp (rs3135506)	CG	12	24.5
	GG	37	75.5
Prothrombin G20210A (rs1799963)	GG	49	100
F5 Arg506Gln (rs6025)	GG	49	100
MTHFR C677T (rs1801133)	CC	13	26.5
	CT	31	63.3
	TT	5	10.2
LIPC C-514T (rs1800588)	CC	19	38.8
	CT	24	49
	TT	6	12.2
LPA I4300M (rs3798220)	TT	47	95.9
	TC	2	4.1
PPAR-alpha L162V (rs1800206)	CC	43	87.8
	CG	6	12.2
APOA5 1131T>C (rs662799)	TT	46	93.9
	TC	3	6.1
APOE Haplotipo APOE2/3/4 (rs429358)	TT	42	85.7
	TC	7	14.3
APOE Haplotipo APOE2,3,4 (rs7412)	TC	7	14.3
	CC	42	85.7

## Limitations

The major limitation of this study was the sample size, which limited us from being able to extrapolate the results to the Spanish population. A larger sample size would result in a sophisticated design and a greater budget, that would limit the number of publications aligned in a deeper analysis.
